# Equity in Orthopaedic Knowledge, Open-Access Trends Among the Top 1,000 Cited Articles in Orthopaedic Surgery

**DOI:** 10.5435/JAAOSGlobal-D-25-00297

**Published:** 2026-09-08

**Authors:** Nicholas J. Pettinelli, Michael Anderson, Madison Blackwell, Arjun K. Reddy, Chad D. Hanson

**Affiliations:** From the Saint Francis Trauma Institute (Dr. Pettinelli), Saint Francis Hospital, Tulsa, OK, and the Oklahoma State University Medical Center (Dr. Pettinelli, Dr. Anderson, Dr. Blackwell, Dr. Reddy, Dr. Hanson), Department of Orthopedic Surgery, Tulsa, OK.

## Abstract

**Background:**

Highly cited articles in orthopaedic surgery are important in shaping practice and education. However, their accessibility through open access (OA) remains unclear despite increasing emphasis on equitable research dissemination.

**Objective:**

The aim of this study was to evaluate the prevalence of open access among the 1000 most-cited publications pertinent to orthopaedic surgery and examine trends by journal, decade, and study design. It was hypothesized that access to most of the most-cited orthopaedic publications is behind paywalls.

**Methods:**

We conducted a cross-sectional bibliometric analysis of the top 1000 most-cited orthopaedic surgery journal articles indexed in Scopus from 1980 to 2025. OA status was determined using the Unpaywall criteria adapted by Scopus, which categorizes publications as gold, green, bronze, hybrid, or closed. Descriptive statistics, chi-square tests, and nonparametric comparisons were used to evaluate OA prevalence across article types, journals, and decades. Citation density and OA status were compared using Mann-Whitney U tests and negative binomial regression.

**Results:**

Among the top 1000 cited articles, 724 (72.4%) were not available through some form of OA. 276 (27.6%) were available through OA. Most open access was tagged as bronze (12.4%) or green (11.3%), while only 3.9% were published in fully OA journals. No hybrid OA articles were identified. OA prevalence varied among top journals, with *Clinical Orthopaedics and Related Research* showing the highest proportion (43.2%) and the *American Journal of Sports Medicine* the lowest (8.1%). OA availability differed significantly by article type (χ^2^ = 15.88, *P* = 0.0032); randomized controlled trials and preclinical studies were more likely to be open than cohort studies. OA prevalence increased significantly over time, from 0% in the 1980s to 63.6% after 2020s. OA articles had higher annual citation densities (28.0 vs. 23.9 citations/year; *P* < 0.0001), although OA status was not a notable predictor of total citations in multivariable models.

**Conclusion:**

Despite positive trends in open access, most influential knowledge in orthopaedic surgery remains closed access, representing a notable barrier to the global orthopaedic community.

Citation analysis is widely used to identify influential works and emerging trends in orthopaedic surgery research. Highly cited publications often reflect consensus-shaping studies, novel surgical techniques, or foundational clinical tools.^1-3^ As the global academic community increasingly embraces principles of open science, the accessibility of these influential works through open-access (OA) publishing has become a key concern. OA offers unrestricted online access to peer-reviewed research, promising faster knowledge dissemination, greater readership, and enhanced visibility, especially in low and middle-income countries.^4-6^

Previous bibliometric analyses have provided detailed profiles of the most-cited orthopaedic surgery articles. In 2011, Lefaivre et al^1^ identified the 100 most-cited articles in orthopaedic surgery, noting that most were clinical studies—primarily case series or classification tool validations—with a predominance of Level IV evidence. A decade later, Lum et al^2^ updated this list and found that citation counts among the top articles had increased by 172%, with topics shifting subtly toward more contemporary areas of focus, such as joint arthroplasty and spine surgery. Other specialty-specific analyses have followed, including an evaluation of the top-cited works published in *Orthopaedics & Traumatology: Surgery & Research* (OTSR), which confirmed that article influence was not necessarily associated with study design rigor.^3^

Despite these insights, relatively little attention has been given to the accessibility of these influential works. Studies in other fields have suggested that a considerable proportion of top-cited articles remain behind paywalls, limiting their utility for students, clinicians, and researchers without institutional access.^7-9^ In orthopaedics, Yammine^7^ found that only 18% to 20% of systematic reviews and meta-analyses related to musculoskeletal care were available through full open access. Similarly, Sabharwal et al^8^ reported that while 32% of orthopaedic journals offered hybrid OA options, only 8% were fully OA; yet, OA status did not seem to affect impact factor or frequency of Level I evidence publication. Franko and Okike^9^ identified 42 English-language OA orthopaedic journals but noted that nearly one-third were potentially predatory, raising concerns about research quality and editorial integrity.

Open access is particularly critical in resource-constrained environments. Doughty et al^10^ demonstrated that surgeons in sub-Saharan Africa frequently relied on OA platforms such as PLoS Medicine because many influential orthopaedic journals were not accessible through global health initiatives such as HINARI. This disparity can have far-reaching consequences for surgical education, guideline development, and evidence-based practice in low-income countries, but also within our own country for those without institutional access.

The relationship between OA publishing and peer-review integrity has also come under scrutiny. Chloros et al^11^ found that 66.7% of orthopaedic journals used double-blind peer review, but a notable proportion lacked transparency in review processes, and many allowed author-suggested reviewers without disclosure of safeguards. Meanwhile, journal-level variation in publication time lines may further influence accessibility and effect because some journals delay full-text availability by months after online acceptance.^12^

Given the increasing emphasis on research accessibility and equity in global surgery, understanding the open-access status of foundational orthopaedic research is both timely and necessary. The objective of this study was to quantify the prevalence and types of open access among the top 1000 most-cited orthopaedic surgery articles and to examine trends by journal and research categories. We hypothesized that most highly cited orthopaedic literature is not available as open access, and that highly cited randomized control trials are the most prevalent content available through open access.

## Methods

### Database Search and Identification of Included Studies

A cross-sectional bibliometric analysis was completed by creating a search query for “Orthopedic Surgery” through the Scopus [Elsevier] database, from years 1980 through July 31, 2025 (Table 1). Methodology was aligned with the BIBLIO checklist (Supplemental Figure 1, http://links.lww.com/JG9/A529). Scopus is a large database tracking the citations and abstract of peer-reviewed literature across many disciplines. Original peer-reviewed journal articles published in English were included in the initial search, yielding s67,743 results (Supplemental Figure 2, http://links.lww.com/JG9/A530). Results from this search were then sorted from high to low by their respective number of citations. Information on the top 1000 cited published articles pertaining to orthopaedic surgery were then extracted and exported to Microsoft Excel Microsoft Corporation (2010; Excel [Computer Software]) for analysis. Information for each published article included title, authors, year, journal, country, DOI, OA status, and number of citations. OA status was assigned according to Unpaywall/Scopus criteria. Gold OA articles are entirely open access and available online, hybrid articles are OA but present in subscription-based journals, bronze OA status signifies material that is temporarily available as open as part of a promotion or special issue, and green OA includes preprints and postprints uploaded by authors to publicly available online repositories. Closed articles are not available through OA in any form. The most common keywords in the extracted abstracts and titles of included studies were aggregated to identify the 10 most common topics found among the 1000 most-cited articles in orthopaedic surgery (Table 2). VOSviewer version 1.6.20 (2023) [Computer Software] was used to create a heat map of the top 1228 keyword co-occurrences in these 1000 most-cited works (Supplemental Figure 3, http://links.lww.com/JG9/A531).^13^

**Table 1:** Search Query Structure for Scopus

Database	Search Query
Scopus	TITLE-ABS-KEY (orthopedic surgery) AND PUBYEAR > 1979 AND PUBYEAR < 2027 AND (LIMIT-TO ( SRCTYPE, “j”) ) AND (LIMIT-TO ( DOCTYPE, “ar”) ) AND (LIMIT-TO ( SUBJAREA, “MEDI”) ) AND (LIMIT-TO (LANGUAGE, “English”))

**Table 2:** Dominant Research Topics Among the 1000 Most-Cited Articles in Orthopaedic Surgery

Rank	Topic (heuristic keyword family*)	No. of articles	% of corpus
1	Hip arthroplasty/hip disorders	264	26.40
2	Fracture fixation/trauma	249	24.90
3	Bone biology/osteoporosis/bone-loss	247	24.70
4	Pain management/analgesia	221	22.10
5	Spine/vertebral pathology and surgery	197	19.70
6	Infection (PJI, sepsis, etc.)	140	14.00
7	Rotator-cuff and shoulder pathology	103	10.30
8	ACL injury/knee-ligament reconstruction	92	9.20
9	Knee arthroplasty (TKA/UKA)	64	6.40
10	Bone and soft-tissue tumors/oncology	40	4.00

Topic assignment was automated using keyword matching in titles + abstracts (eg, “hip,” “acetabular,” “femoral” → hip-related and “fracture,” “fixation,” etc. → trauma). A single article could match multiple topic families; counts reflect at least one match per family.

### Data Analysis

Data was entered and analyzed using Jamovi cloud-based open access statistics The Jamovi project (2024). Jamovi. (Version 2.6) [Computer Software].^14^

Of the top 1000 search, the top five journals were ranked according to aggregate citations. Proportional statistics were used to show the breakdown of open-access availability by journal. The most recently recorded impact factor for each of these journals was reported. A Spearman rank correlation was used to determine whether journal rank by citations was correlated to the number of open-access articles contained within the top 1000 cited orthopaedic surgery articles.

Of the exported top 1000 cited articles, the Scopus database had assigned research categories to each of the articles. Overall, 47 of 1000 articles were originally unclassified by Scopus on data extraction and were manually assigned a category of research after consensus by two authors. The proportion of open access by research category was also recorded. A Chi-square test with Cramer V for effect size was run to determine whether certain article categories such as RCTs were more likely to be open access than others. Subsequently, a Chi-square omnibus test was used to complete a pairwise 1:1 comparison of article types and their probability of being open access.

A logistic regression was used to determine whether open access has evolved by decade. A Mann-Whitney U analysis was run to determine whether open-access articles receive more citations than closed articles.

## Results

### Open-Access Prevalence in Top-Cited Literature

A total of 1000 top-cited orthopaedic surgery journal articles were extracted from the Scopus query, as given in Table 1. The included articles all had at least 180 citations. The most cited article was “Projections of primary and revision hip and knee arthroplasty in the United States from 2005 to 2030” by Kurtz et al^15^, cited 7266 times. Regarding topics, hip reconstruction, trauma fixation, and bone biology/osteoporosis dominate the most highly cited orthopaedic literature. Each of these categories possessed roughly a quarter of the top 1000 citations (Table 2). Knee arthroplasty and bone and soft-tissue tumors/oncology were less prevalent in the top 1000 cited articles.

Among the 1000 most highly cited articles in orthopaedic surgery, 72.4% were not available through open access (Table 3). 27.6% of articles were available through some level of open access, with 3.9% of total articles available through gold OA.

**Table 3:** Distribution of Level of Open Access in 1000 Most-Cited Orthopaedic Surgery Articles

OA Category	Count	Percent (%)
Closed (no OA)	724	72.4
Bronze	124	12.4
Green	113	11.3
Gold	39	3.9
Hybrid	0	0

### Top Orthopaedic Journals

**Figure 1:**
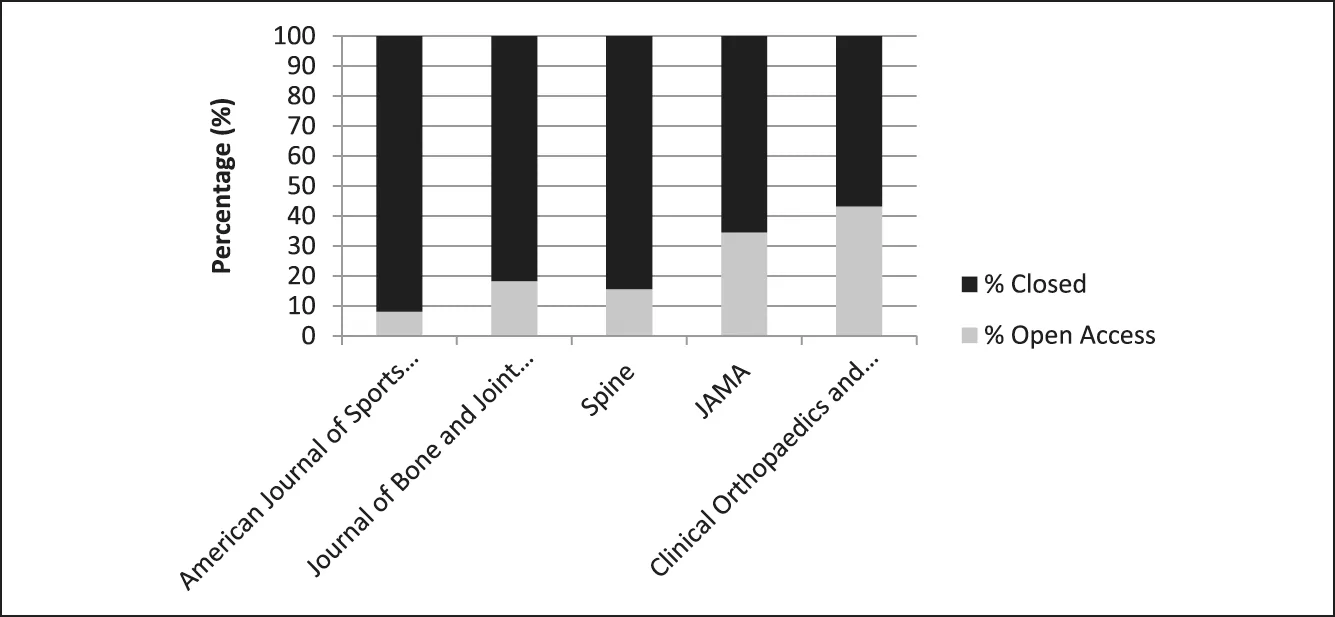
Chart demonstrating the proportion of open-access vs. closed-access top 1000 articles by highly cited orthopaedic surgery journals.

The top five orthopaedic surgery journals in terms of aggregate citations were the American Journal of Sports Medicine, the Journal of Bone and Joint Surgery, Spine, JAMA, and the Journal of Clinical Orthopaedics and Related Research (Table 4). The flagship sports medicine journal AJSM has the lowest proportion of OA content, with 8.1% of highly cited articles available through open access (Figure 1). Clinical Orthopaedics and Related Research shows the highest proportion of publicly available content in this cohort, with 43.2% of highly cited publications available through OA (Table 4). Journals with a higher citation rank showed a nonsignificant weak tendency to have slightly fewer open-access articles, through Spearman rank correlation (rho = −0.2, *P* = 0.75).

**Table 4:** Top 5 Orthopaedic Surgery Journals Ranked by Total Citations Within the Top 1000 Data Set and Prevalence of Open Access

Ranked Journal by Aggregate Citations	Total Citations	Total Articles Within Top 1000	Open-Access Articles	% Open Access	Closed/Non–Open-Access Articles	% Closed
*American Journal of Sports Medicine*	39327	135	11	8.1	124	91.9
*Journal of Bone and Joint Surgery*	38132	126	23	18.3	103	81.7
*Spine*	25485	109	17	15.6	92	84.4
*JAMA*	13429	29	10	34.5	19	65.5
*Clinical Orthopaedics and Related Research*	11303	37	16	43.2	21	56.8

### Categories of Research

**Figure 2:**
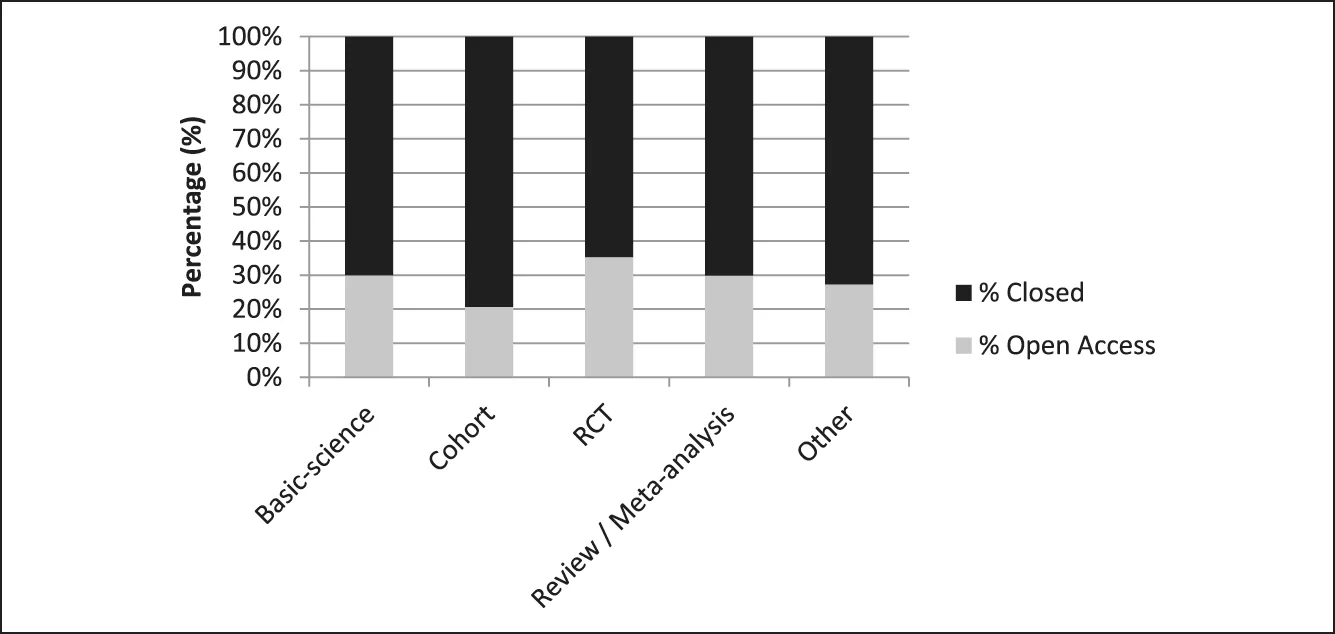
Chart demonstrating the proportion of open-access vs. closed-access research articles by category of research.

The categories of research articles in the top-cited orthopaedic literature is given in Table 5, indicating the prevalence of open access per category. An overall χ^2^ test (χ^2^ = 15.88, df = 4, *P* = 0.0032, Cramer V = 0.13) demonstrated that the distribution of open-access availability differs markedly across article categories, but the association is weak. Preclinical and randomized control trials demonstrated higher proportions of OA (∼30 to 35%) compared with cohort studies (∼21%) (Figure 2). Post hoc pairwise analysis compared OA rates between different research categories. RCT studies were significantly more likely to be open access than cohort studies (*P* < 0.05), and cohort articles were significantly less likely to contain open-access material than preclinical studies (*P* < 0.05). All other pairing showed no significant difference after correction.

**Table 5:** Distribution of Top 1000 Cited Articles in Orthopaedic Surgery by Category of Research Provided by Scopus Database

Article Category	Closed Access	Open Access	Row Total
Basic science/laboratory/cadaver	232	99	331
Cohort/observational/case series	291	76	367
RCT	132	72	204
Review/meta-analysis	61	26	87
Other (a few articles not fitting main 4 groups)	8	3	11
Column totals	724	276	1000

A total of 47 articles were originally unclassified by Scopus on data extraction and were manually assigned a category of research after consensus by two authors.

### Open Access in Articles Published Over Recent Decades

**Figure 3:**
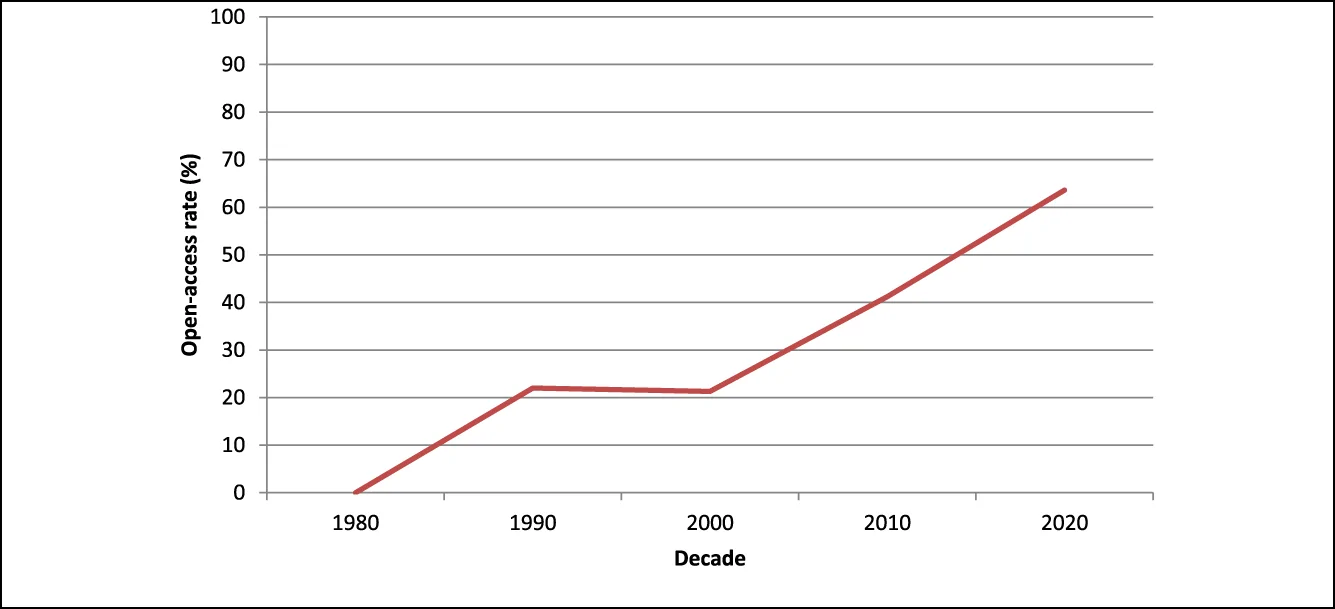
Chart demonstrating the trend of open access by decade of publication year.

We analyzed the prevalence of OA material by decade of publication (Figure 3). A logistic regression model (OA = 1 vs 0) with decade of publication as a continuous predictor showed a statistically significant upward trend in open-access prevalence by decade. Each 10-year increase in publication date was associated with an 8% increase in the odds that an article was open access (adjusted odds ratio = 1.08, 95% CI, 1.05 to 1.10, *P* < 0.0001). The model attributed a small amount of the variability in OA prevalence to decade alone (McFadden R^2^ = 0.047).

### Open Access and Citations Over Decades

Of the 1000 most-cited orthopaedic surgery articles, 276 (27.6%) were available through OA. Raw citation counts did not differ significantly between OA and closed-access articles (median 352 [IQR 208-641] versus 322 [189-594]; Mann-Whitney *U* = 107,449; *P* = 0.065; effect size *r* = 0.06). Annualized citation density defined as citations per year since publication was significantly higher among OA articles (median 28.0 [17.1-45.0] versus 23.9 [14.7-38.6]; Mann-Whitney *U* = 124,109; *P* = 3.1 × 10^−9^; *r* = 0.19). However, in a negative-binomial regression controlling for publication year, OA status was not a statistically significant predictor of citation count (incidence rate ratio 1.07; 95% CI, 0.93-1.24; *P* = 0.34).

## Discussion

The main finding of this cross-sectional bibliometric analysis is that only 27.6% of highly impactful research in the field of orthopaedic surgery was freely accessible through OA. The remaining 72.4% of top 1000 cited publications were closed access or behind a paywall and inaccessible to nonsubscribers/those without institutional access. Among OA articles, most were either bronze (12.4%) or green (11.3%), with a minority published in fully OA journals (3.9% gold OA). No articles in the top 1000 were available as hybrid access. These findings align with earlier estimates by Yammine, who reported that only 18 to 20% of musculoskeletal systematic reviews were available through full OA,^7^ and by Sabharwal et al.,^8^ who noted that only 8% of orthopaedic journals were fully OA. This study highlights a lack of open access, particularly in articles that should be widely available to the orthopaedic community.

This analysis expands on previous literature by providing a large-scale, citation-focused snapshot of OA availability in high-impact orthopaedic surgery research. Compared with the 18%-20% OA prevalence reported in previous specialty-focused studies, our observed rate of 27.6% may reflect both a broader inclusion of article types and rising OA trends over time. For instance, this decade-wise analysis showed that OA prevalence increased from 0% in the 1980s to 41.2% in the 2010s, and to 63.6% for highly cited articles published since 2020. Furthermore, unseen variables such as journal-specific policies, funding, and the general volume of research production are examples of other factors that can be responsible for this trend. The trend observed in this study is consistent with global publishing patterns described by Suber^4^ et al, where mandates from funders and institutions, along with cultural shifts toward open science, have gradually improved research accessibility. With the shift of increasing open access, the proliferation of low-quality, predatory OA journals has also complicated the landscape for those seeking credible, accessible research.^9^ The addition of the Directory of Open Access Journals (DOAJ) has emerged as a registry to verify nonpredatory open-access journals.^16^

This study also found substantial variation in OA availability across journals. Clinical Orthopaedics and Related Research had the highest OA proportion (43.2%), while more highly cited journals such as the American Journal of Sports Medicine (AJSM) and the Journal of Bone and Joint Surgery (JBJS) had OA rates of just 8.1% and 18.3%, respectively. This heterogeneity echoes some findings by Pfeil et al^17^ who highlighted that citable open-access articles did not vary markedly between “Big 5” publishers and “Non Big 5” publishers. The data presented here support an interpretation that journals with higher aggregate citation counts tended to have slightly lower OA proportions, although the correlation was not statistically significant.

Regarding study design, randomized controlled trials (RCTs) and preclinical studies were significantly more likely to be open access than cohort studies (35% and 30% vs. 21%, respectively; *P* = 0.0032). Pairwise comparisons showed RCTs had higher OA prevalence than cohort studies (Bonferroni-adjusted *P* < 0.001). Cohort studies were found to have the highest proportion of closed access (79%). These findings are encouraging in the context of equitable access to quality research because randomized controlled trials seem to drive the most direct changes in patient care in the field of orthopaedics.^18-20^ RCTs that compare surgical with nonsurgical care, large trauma trials, and analyses of perioperative interventions have most directly changed orthopaedic practice, while registry and observational studies have helped contextualize these changes and highlight gaps in implementation.^21-23^ One potential explanation of our findings is that OA is beginning to be driven by funder mandates that prioritize OA for interventional and preclinical research. Similar patterns were observed by Chloros et al,^11^ who noted that editorial transparency and peer-review practices in orthopaedic journals vary by study type and may influence OA decisions.

While raw citation counts between OA and closed-access articles did not differ significantly (352 vs. 322, *P* = 0.065), OA articles demonstrated significantly higher annualized citation densities (28.0 versus 23.9 citations/year; *P* < 0.000001). This citation benefit is consistent with the findings of Lynch et al,^24^ who reported greater citation rates for OA articles in impactful journals within spine research. However, Zhang et al^25^ noted an advantage in citations with standard publication versus OA in orthopaedic surgery. In this study, when negative binomial regression adjusted for publication year, OA status alone was not an independent predictor of total citation count. This seems to support the conclusions in total ankle arthroplasty literature, where OA articles gained more social media attention but not more citations.^26^ Together, these findings suggest that OA enhances immediate dissemination and online attention but may not uniformly increase long-term citation accrual.

### Open-Access Publishing, Far from Perfect

Although the authors of this study are in support of free information dissemination, the open-access model is not without its flaws. Although well intentioned, in its current state, OA policies have given rise to a “pay to play” culture through APCs. Importantly, there were no hybrid OA publications among the 1000 most cited articles. This is notable, given the widespread availability of hybrid options in subscription journals. Pfeil et al^17^ showed that APCs for hybrid OA articles in orthopaedic journals can exceed $3,000, while APCs in fully open-access journals are roughly $1000 lower.

Integral to the problem is that researchers and academics are both the creator and consumer of intellectual materials, with payment barriers present on both sides of published research. Thus, when given the choice, authors without additional funding are possibly deterred and precluded from choosing to publish through the OA route, thus creating a sort of “submission bias.”^17,27,28^ Furthermore, citation-based metrics contributing to journal prestige such as impact factor and H-index remain dominant in academic promotion, potentially incentivizing publication in standard subscription-based journals.^29^ As mentioned previously, the emergence of predatory OA journals should also encourage authors to do their due diligence in vetting of OA journals. While there are no open-access journals in medicine that match the quality of powerhouse journals such as the New England Journal of Medicine or JAMA, writers are encouraged to consider in the order Leopold (2020) has laid out—quality of the publication process, visibility, cost, prestige, and speed when thinking about where they should publish.^27,30^

### Implications for Equity in Education, Training, and Patient Care

The potential implications of restricted access are notable. As others have emphasized, limited access to foundational orthopaedic literature disproportionately affects surgeons, students, residents, and researchers in resource-constrained environments.^10^ However, it is important to note that restricted access not only affects those abroad but also directly limits resources available to those lacking the infrastructure and financial support of a large academic center.

The field of orthopaedic surgery relies heavily on previous research as well as classification systems to categorize conditions and guide diagnosis, treatment, and surgical interventions.^31^ As students and residents pursue a rigorous career in orthopaedic surgery, they learn these systems, which are largely based on landmark research articles. Consistent with this cross-sectional bibliometric analysis, these original articles are rarely accessible through OA and thus learners are deprived of the context behind these important works that regularly dictate diagnosis and treatment.

One potential work-around for gaining access to restricted articles is through student/resident/faculty request via interlibrary loans. However, these processes usually take several days to process and represent an undesirable delay in access to information. In an age where knowledge and information is at our fingertips, it is counterproductive that access to landmark articles, including clinical practice guidelines, recommendations, and foundational information important to patient care, is limited. Perhaps, one of the more progressive approaches to equitable dissemination of foundational material by journals has been making their content freely available on public repositories such as PubMed Central after an embargo (subscription-only) period.^27^ The journal Clinical Orthopaedics and Related Research (CORR) enacts this practice, which is reflected in this study where 43.2% of highly cited content was available through OA. With these factors in mind, restricting access to this information ultimately may affect patients and the ability of orthopaedic surgeons to provide the most evidence-based care.

### Recommendations for Improving Open Access

The findings in this study show that many of the most influential orthopaedic articles remain behind paywalls, underscoring the need for reform, not just in publishing models but also in journal policies and funding infrastructure. The authors posit several actionable recommendations to improving open-access models. First, funding agencies that support orthopaedic research should prioritize funding for OA publishing. As suggested by others, this can include providing grants to help cover the cost of APCs for researchers in developing regions.^32^ The authors also support the use of OA mandates by funders, such as the NIH Public Access Policy, which recently required that studies completed with NIH funding be submitted to PubMed Central repositories on acceptance for publication.^33^ Second, professional societies should encourage journals to adopt fully OA models and hybrid models that allow authors to choose OA without exorbitant fees that remain a barrier, with a greater push for subsidized fees for those in low-resource settings.^8^ Third, researchers should not be punished or prohibited from publishing their work in peer-reviewed journals for attempting to increase research dissemination through green OA practices, such as uploading their works to preprint databases. However, it is important that we encourage the development of proper safeguards and guidelines by professional societies for interpreting and ensuring scientific rigor in green OA publications.^34^ As mentioned above, embargo periods help journals strike a balance between holding the interest of their subscriber base and making dissemination of research and knowledge equitable.

At its core, open-access publishing can help ensure equitable access to scholarly publication regardless of resources, institution, or country of residence.^35^ Open-acccess publishing may also ensure that relevant research is not left out of the synthesis of best practices through aggregate data in systematic reviews and meta-analyses.

### Limitations

This study has several limitations. Scopus was used for article identification and citation counts, while comprehensive, alternative databases such as Web of Science or Google Scholar may yield different rankings. OA classification was based on Unpaywall API data and publisher-provided metadata, which may misclassify green or bronze OA articles, especially older ones. Finally, citation count, although widely used, does not fully capture research quality or societal influence.

## Conclusion

Despite a rising trend in open-access publication, nearly three-quarters of the most cited orthopaedic surgery articles remain behind paywalls. Increasing open access in orthopaedic research is not just about increased citations but about ensuring equitable access to knowledge that can drive improvements in global surgical practices and patient care. By aligning the efforts of journals, funders, and professional societies, the orthopaedic community can take meaningful steps toward ensuring that high-quality research is more accessible.

## Supplementary Material
