# Supplementary material for: Equity in Orthopaedic Knowledge, Open-Access Trends Among the Top 1,000 Cited Articles in Orthopaedic Surgery
Source: J Am Acad Orthop Surg Glob Res Rev. 2026 Sep 8;10(9):e25.00297. doi: 10.5435/JAAOSGlobal-D-25-00297 (PMC13557761; doi:10.5435/JAAOSGlobal-D-25-00297)
Supplement: Supplementary file 2 [file jagrr-10-e25.00297-s002.docx]

**Supplemental Figure 2.** Flowchart depicting criteria for record inclusion in study, adapted from PRISMA 2020 flow diagram.^21^
