# Supplementary material for: Equity in Orthopaedic Knowledge, Open-Access Trends Among the Top 1,000 Cited Articles in Orthopaedic Surgery
Source: J Am Acad Orthop Surg Glob Res Rev. 2026 Sep 8;10(9):e25.00297. doi: 10.5435/JAAOSGlobal-D-25-00297 (PMC13557761; doi:10.5435/JAAOSGlobal-D-25-00297)
Supplement: Supplementary file 3 [file jagrr-10-e25.00297-s003.docx]

**Supplemental Figure 3.** Keyword coccurrence heatmap generated from top-1000 most cited publications in orthopedic surgery via VOSviewer [computer software].^13^
